# Gender Effect on Political Leaders’ Nonverbal Communicative Structure during the COVID-19 Crisis

**DOI:** 10.3390/ijerph17217789

**Published:** 2020-10-24

**Authors:** Tsfira Grebelsky-Lichtman, Roy Katz

**Affiliations:** 1 Department of Communication and The Program of Conflict Research, Management and Resolution, The Hebrew University, 91905 Jerusalem, Israel; roy.i.katz@gmail.com; 2 Department of Education, Ono Academic College, 55000 Kiryat Ono, Israel

**Keywords:** gender, nonverbal communication, COVID-19, leadership, political communication

## Abstract

During the COVID-19 pandemic, there has been intense interest in political leaders’ nonverbal communicative structures (NCS) during televised appearances. This study analyzes the effect of gender on leaders’ NCS and presents theoretical and analytical frameworks of gendered NCS. We analyzed 20 televised appearances by 10 heads of state (five males and five females) from democratic Western countries during the COVID-19 pandemic. The findings revealed that gender had a significant effect on leaders’ NCS, indicating that leaders presented NCS that corresponded to their gender. Male leaders’ masculine NCS included competition, warning, threatening, and scaring behavior, broad proxemics, tension leakage, and illustrative gestures, while female leaders presented feminine NCS of cooperativeness, emotional communication, empathy, optimism, eye contact, and flexible expressions. Furthermore, the effect of gender on leaders’ NCS had an interaction effect with the situation of the pandemic, indicating that countries with a female leader had fewer diseased and severe cases and more calmness and healing NCS. The conclusions present theoretical and analytical frameworks that explain the central effect of gender on contemporary leaders’ NCS. This study develops advanced distinctive profiles for male versus female leaders’ NCS of emotions, cognition, and behavior during a crisis.

## 1. Introduction

Nonverbal communicative structure (NCS) has a central role in perceptions of politicians’ leadership, charisma, confidence, and trust [1]. Leaders’ NCS is essential in affective communication, influence, and persuasion, especially during crisis and challenging periods of stress, fear, and uncertainty [2] (Wicks et al., 2017). During crisis, people are highly sensitive to a leader’s NCS. This study analyzes gender effect on political leaders’ NCS in televised appearances during the COVID-19 crisis and develops a gender perspective of political communication that delineates gender differences in emotions, cognition, and behavior.

The recent global health crisis has forced political leaders to face their people urgently through official televised COVID-19 appearances, to guide, inform, calm, and support. Leaders’ NCS during televised COVID-19 appearances express contemporary processes in the political communication of personalization [3,4] and emotionalism [5,6], which highlights the importance attributed to NCS.

NCS is strongly correlated with gender and affect evaluations of male/female political leaders [7]. Political personalization and emotionalism emphasize leaders’ gender identity [8], which leads to the emergence of societal expectations of gendered features and behaviors [9,10]. The societal gender expectations of male and female leaders defined nonverbal patterns that have been indexed as masculine or feminine in political communication [11], and utilized gendered leadership styles. Deviation from societal gender expectations and behavioral norms may be socially punishable [12] and cause a “backlash” effect of negative perceptions and evaluations of the leader [13]. This study is the first to analyze the gender effect on political leaders’ NCS during a pandemic crisis. Therefore, the objective of the current study is to expose the effect of gender on leaders’ NCS during the COVID-19 crisis (see RQ1 on Table 1).

The theoretical framework of this study is grounded in the role-congruity theory [14], which argues that female politicians face incongruity between the female gender role based on feminine societal expectations and leadership roles based on masculine societal expectations. According to the role-congruity theory, the effect of gender on leaders’ behavior is especially important because the political sphere is arguably a masculine space. Moreover, the political arena favors politicians who display masculine characteristics [15]; thus, female politicians who wish to succeed must express masculine NCS [16].

Previous analyses of male versus female political leaders have found that most gender difference perceptions pertain to communal and agentic attributes [17]. Empirical evidence delineated the type of nonverbal manifestations associated with gender and leadership and presented the psychological correlates associated with masculine and feminine nonverbal expressions [18].

Masculine agentic characteristics of political leaders primarily describe four dimensions of assertiveness, being controlling, confidence, and rational tendencies [14]. The psychologically correlated nonverbal manifestations of assertiveness include aggressive, sharp movements that express an unequivocal, determined message, and clenched fists [19]. As for the second dimension of control, the psychologically correlated nonverbal manifestations, including broad proxemics that express aggressive behavior by taking up a lot of space, indicating control, may signal threatening conduct or frightening behavior of leaders with high status and self-confidence [20]. The psychologically correlated nonverbal manifestations of the third dimension of confidence include dominant, forceful, ascending postures, such as a very straight and upright body posture, conveying confidence and stability which is attributed to perceptions of leadership, charisma, and determination [2]. Regarding the fourth dimension of rationality, the psychologically correlated nonverbal manifestations include rational–intentional illustrative gestures that improve understanding, memory, involvement, and increase the impression of vitality and reciprocity of engagement [7].

Feminine communal characteristics of political leaders primarily describe four dimensions: emotional communication, interpersonal sensitivity, kindness, and empathy [14]. The psychologically correlated nonverbal manifestations of emotional communication include an expressive voice of affection, which exposes their emotional state and presents affective communication that encourages listening, influences perceptions of trustfulness, and enhances personal connections [19]. As for the second dimension of interpersonal sensitivity, the psychologically correlated nonverbal manifestations include making eye contact, which is important for increasing credibility and trust and for political personalization, as well as affective communication, emotionalism, and even liking [21]. The psychologically correlated nonverbal manifestations of the third dimension of kindness include extensive facial expressions, such as smiling, which expresses optimism and plays an important role in perceptions of kindness, calmness, and an ability to withhold pressure. Smiling also plays a central role in increasing engagement and cooperation [20]. Gentle, round, and small movements are also correlated to perceptions of kindness [1]. Regarding the fourth dimension of rationality, the psychologically correlated nonverbal manifestations of empathy include diverse intonation, which represents concern of the welfare of other people, mainly by a tone of compassion that increases involvement and enhances understanding, memory, and mutual engagement [22].

Based on our theoretical framework and these empirical evidences, the second objective of the current study is to analyze whether female vs. male leaders expressed feminine or masculine NCS during the COVID-19 crisis (see RQ2 on Table 1).

During a crisis such as COVID-19, leaders’ NCS may be affected by the situation of the pandemic. When the situation of the pandemic is minor (that is, with few diseased and severe cases), the leader’s NCS may display nonverbal expressions of confidence, certainty, control, and optimism. However, when the situation of the pandemic is severe (when there are many diseased and severe cases), the leader’s NCS may express fear, helplessness, uncertainty, and nonverbal leakage of stress and tension. Nonverbal leakage of stress occurs when concealed information is displayed involuntarily [23,24]. Nonverbal leakage has inhibitive emotional, cognitive, social, and political implications, which affects politicians’ perceived credibility and trustworthiness [25]. During periods of crisis, viewers are sensitive to nonverbal leakage of tension in political leaders’ conduct. In particular, the TV close-ups of the leaders’ faces emphasize tension leakage and increase the impact of these movements. Therefore, the situation of the pandemic and the level of severity of the crisis may have a mediated effect on gender and leaders’ NCS. Hence, the third objective of the current study is to expose the interaction effect of the situation of the pandemic on gender and leaders’ NCS during the COVID-19 crisis (see RQ3 on Table 1).

### Research Questions

We have advanced the following research questions, summarized in Table 1:

## 2. Methods

### 2.1. The Corpus of the Study

This study analyzed 20 televised appearances by 10 political leaders (two appearances for each) during the COVID-19 crisis (mean length was 23:30 min, SD = 8:30). As can be seen in Table 2, all of the leaders (five of whom were men and five of whom were women) were prime ministers/presidents from democratic Western countries (the corpus of the study contains leaders from democratic Western countries, all of which actively promote transparency and independent media. The primary aim was to compare methodologies of leaders’ NCS based on gender: female leaders (FL) vs. male leaders (ML); thus, we aimed to control other factors when constructing the FL group and the ML group. Age factor; the corpus contains young leaders (FL New Zealand’s PM Ardern, 39, and ML Trudeau, Canada, 48) and older leaders (FL Germany’s Merkel 65, and ML US President Trump, 73). The experience factor; the corpus includes experienced leaders (FL Merkel, who has led Germany since 2005, and ML Israel’s PM Netanyahu since 2009), as well as newcomers to the role (FL Mette Frederiksen in Denmark since 2019, and ML Italy’s Giuseppe Conte since late 2018). The political wing factor; the corpus includes left wings (FL of Belgium and ML of Canada), as well as right wings (ML of US and FL of Germany). The factor of type of democracy; the corpus includes federal parliamentary democracy (e.g., Belgium with FL and UK with ML), constitutional monarchies (e.g., Canada with ML and New Zealand with FL) and parliamentary republics (of Finland with FL and Italy with ML)).

### 2.2. Coding System and Intercoderliabilities

The analysis of leaders’ NCS was a multi-tiered system for observed nonverbal communication classification, which included gestures, postures, facial expressions, vocalic characteristics, and performance. As can be seen in Table 3, we analyzed political leaders’ gendered NCS with an analytical framework of masculine and feminine NCS patterns; for each NCS pattern, we conducted intercoder reliability using Cohen’s Kappa. Two undergraduate research assistants, each trained for approximately nine hours, coded the NCS. In establishing inter-coder reliability, disagreements between coders were resolved by clarifying and then reapplying the coding book guidelines (see Appendix A). As can be seen in Table 3, reliability was high, ranging from 0.87 to 0.94.

### 2.3. Procedure and Data Analyses

In order to analyze the effect of gender on leaders’ NCS (RQ1), we conducted multiple regression analysis. The dependent variables constituted of leaders’ NCS, which was operationally constructed as a sum of the frequencies of the NCS patterns (detailed in Table 1). The independent variable was the gender of the political leader. Moreover, in order to analyze the masculine and feminine NCS of male versus female leaders (RQ2), boxplots were employed for the extreme maximum and minimum values, the lower and upper quartiles, and the average of each masculine/feminine NSC pattern. Furthermore, for analyzing the interaction effect of leaders’ gendered NCS with the situation of the pandemic (RQ3), we computed multiple regression analysis. This regression analysis contained the variables of gender and leaders’ NCS that were examined in RQ1 combined with the variable of the situation of the pandemic, which was operationalized as the ratio of death/diseased cases.

## 3. Results

### 3.1. The Effect of Gender on Political Leaders’ NCS

Regarding RQ1, multiple regression analysis found that gender had a significant main effect on political leaders’ NCS (β = 2.31, B = 134.21, t = 5.17, *p* < 0.001). Male leaders’ NCS was significantly different from that of female leaders. Female leaders presented significantly distinctive NCS compared to male leaders during the COVID-19 crisis.

**Figure 1:**
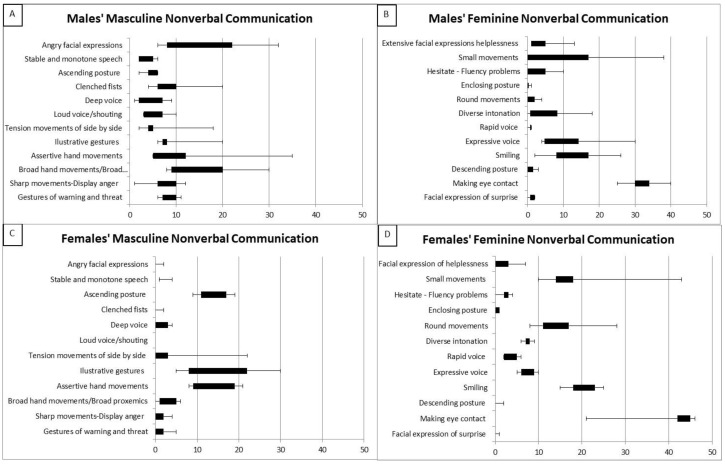
(**A**–**D**) Male vs. female political leaders’ masculine and feminine nonverbal communicative structure (NCS). (Note. Figure 1A–D illustrate political leaders’ gendered nonverbal communicative structure. Boxplot displays the extreme maximum and minimum values, the lower and upper quartiles, and the average. (**A**). Males’ masculine nonverbal communication. (**B**). Males’ feminine nonverbal communication. (**C**). Females’ masculine nonverbal communication. (**D**). Females’ feminine nonverbal communication.).

As for RQ2, political leaders presented conduct that tended to correspond to their gendered NCS. Thus, male political leaders’ NCS displayed significantly more masculine patterns (*M* = 8.53; *SD* = 2.04) than feminine patterns (*M* = 7.32; *SD* = 3.42); Multiple regression analysis of masculine NCS revealed a main effect for gender (β = 1.36, B = 122.61, t = 4.026, *p* < 0.005), indicating that male leaders tended to express more masculine NCS than female leaders. By contrast, female political leaders’ NCS displayed significantly more feminine patterns (*M* = 9.08; *SD* = 0.42) than masculine (*M* = 5.72; *SD* = 3.04). Multiple regression analysis of feminine NCS revealed a main effect for gender (β = −0.96, B = −83.85, t = −3.78, *p* < 0.005), indicating that male leaders tended to express less feminine NCS than female leaders. Furthermore, the findings of feminine and masculine NSC (see boxplots analyses, Figure 1A–D) delineated the gendered NCS of female vs. male political leaders during COVID-19 televised appearances.

### 3.2. Male Leaders’ Gendered NCS

Male political leaders’ NCS during the COVID-19 crisis contained mainly masculine patterns (Figure 1A). The main characteristics of male leaders’ masculine patterns were broad hand movements of broad proxemics (*M* = 16.2; *SD* = 9.07); for example, Johnson (Photo 1) and Netanyahu (Photo 2).


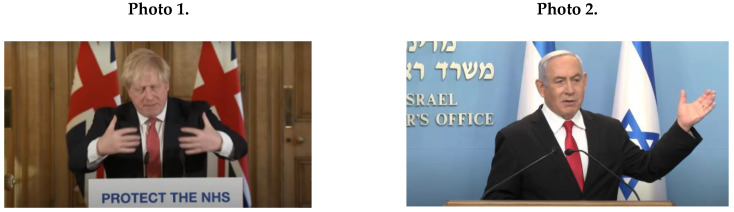


Male leaders’ masculine NCS included angry facial expressions (*M* = 16.2; *SD* = 8.97), as in Photo 3 (Trump); and clenched fists (*M* = 9.4; *SD* = 6.31); see Johnson (Photo 4) and Netanyahu (Photo 5).


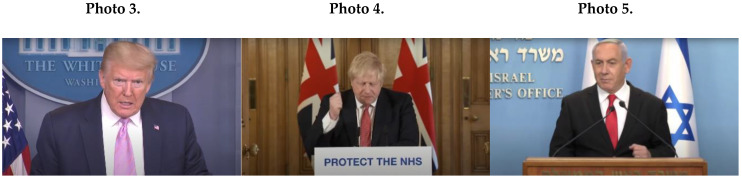


As Figure 1A indicates, male political leaders’ masculine NCS contained sharp movements (*M* = 7.2; *SD* = 4.21) and anger (see Photo 6 of Trump using a sharp beating gesture). Furthermore, male leaders’ behavior contained masculine assertive gestures of warning and threatening (*M* = 8.4; *SD* = 2.07). In Photo 7, for example, Trump used a finger-pointing gesture of warning and threat, combined with an angry and threatening facial expression.


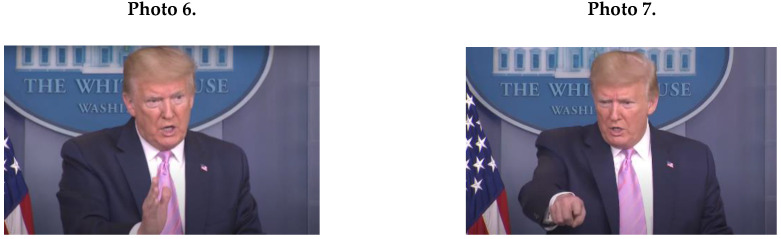


An important masculine NCS of male leaders was tension leakage of side-by-side movements and licking of lips (*M* = 4.5; *SD* = 0.71), which contradict messages of calmness, ease, confidence, and control (see Photo 8 of Trump, showing tension leakage through licking of lips).


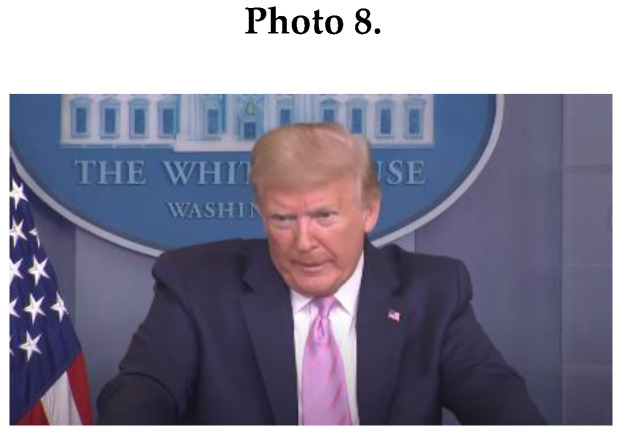


Furthermore, male leaders expressed masculine NCS of illustrative gestures (*M* = 9.8; *SD* = 5.76), for example, see Photo 9 (Johnson) and Photo 10 (Netanyahu).


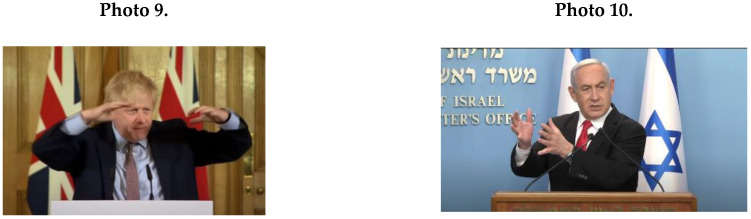


All of the male political leaders’ NCS included a masculine appearance by dressing formally in a suit and tie.

Male political leaders also exhibited few feminine NCS characteristics during televised appearances of the COVID-19 crisis (see Figure 1B). The main feminine pattern was making eye contact (*M* = 32.2; *SD* = 5.5), as presented in Photo 11 of Trudeau. Interestingly, all political leaders were tied to their notes, which were written in advance and presumably because of the sensitive period of the pandemic and its strong financial implications, the leaders wanted to be accurate, intentional, and prepared (see Photo 12 of Trudeau).


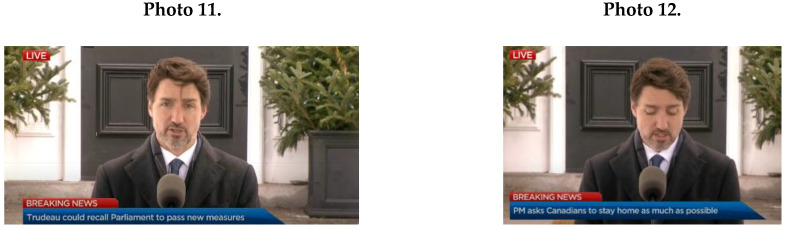


As Figure 1B shows, other feminine NCS exhibited by male leaders involved a descending posture (*M* = 6.0; *SD* = 4.5); for example, Conte (Photo 13) and Trump (Photo 14).


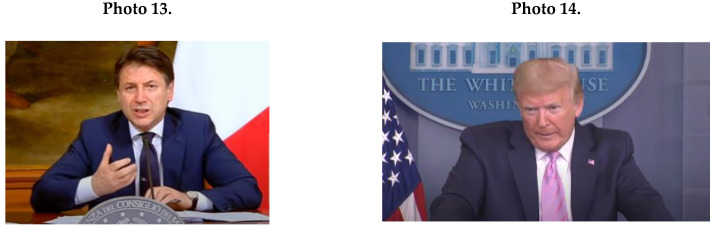


### 3.3. Female Leaders’ Gendered NCS

Female political leaders’ NCS during the COVID-19 crisis contained mainly feminine patterns. Figure 1D shows that the main characteristics of female leaders’ conduct were smiling (*M* = 24.0; *SD* = 4.09); for example, Merkel (Photo 15), Marin (Photo 16), and Ardern (Photo 17). Female leaders generally used extensive facial expressions (see Ardern in Photo 18).


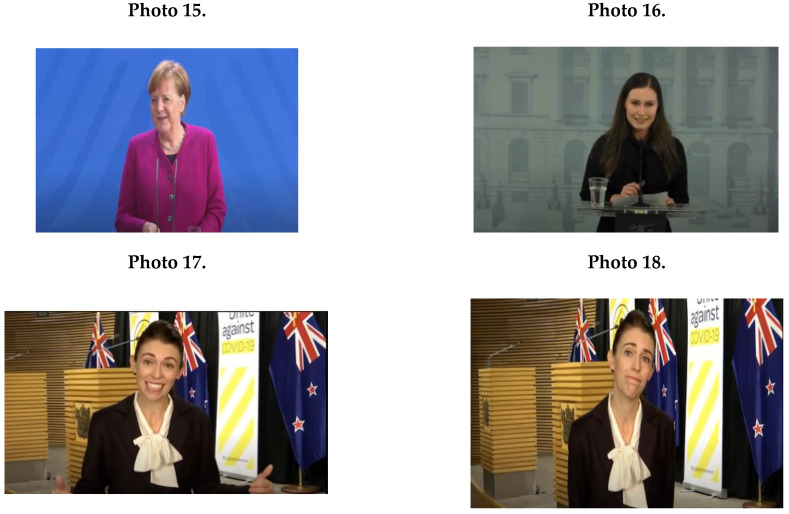


Female political leaders’ NCS mainly contained an effective feminine pattern of making eye contact (*M* = 39.6; *SD* = 10.5). Interestingly, all female political leaders managed to combine being tied to their script text with maintaining eye contact, as shown by Wilmès (Photo 19) and Marin (Photo 20).


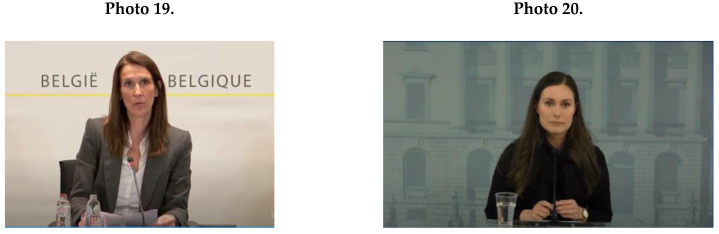


As Figure 1D shows, other feminine NCS displayed by female leaders were round hand movements (*M* = 15.2; *SD* = 7.85) (see Merkel in Photo 21), and small movements (*M* = 20.2; *SD* = 13.08). For example, Frederiksen (Photo 22) used small gestures combined with an enclosing posture.


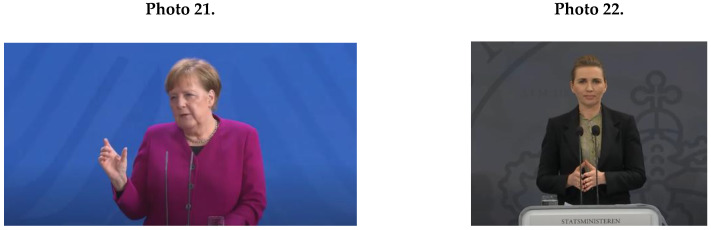


Regarding feminine vocalic aspects, female leaders expressed diverse intonation (*M* = 9.0; *SD* = 6.4). Additionally, female leaders used an expressive voice (*M* = 7.6; *SD* = 2.7). Female political leaders’ NCS during the COVID-19 crisis also contained few masculine patterns. As Figure 1C shows, female leaders displayed an ascending posture (*M* = 16.0; *SD* = 9.6) by using a very straight, upright body posture (Frederiksen in Photo 23, for example).


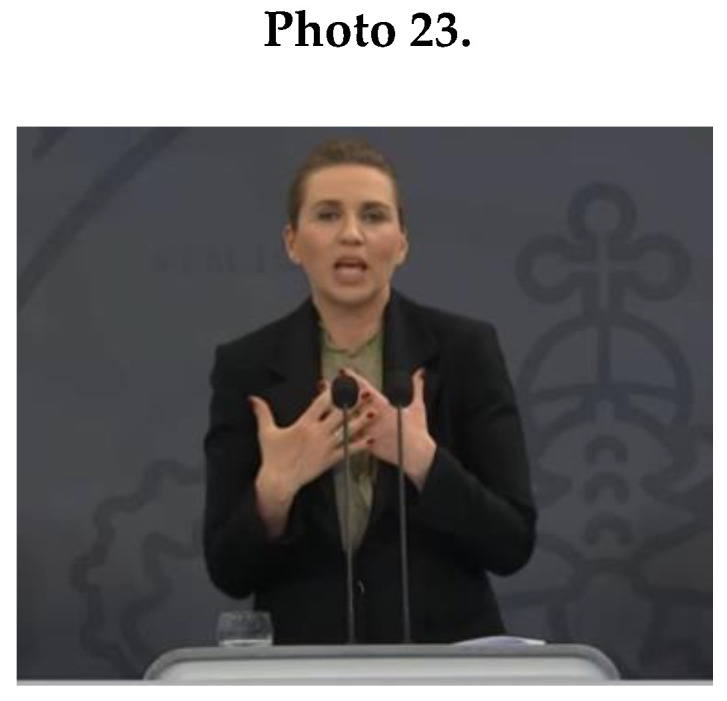


Female leaders combined their NCS with masculine patterns of illustrative gestures (*M* = 14.8; *SD* = 8.6), as well as broad hand movements of broad proxemics (*M* = 3.4; *SD* = 1.7) (see Ardern, Photo 24). Ardern also used an illustrative gesture in Photo 25.


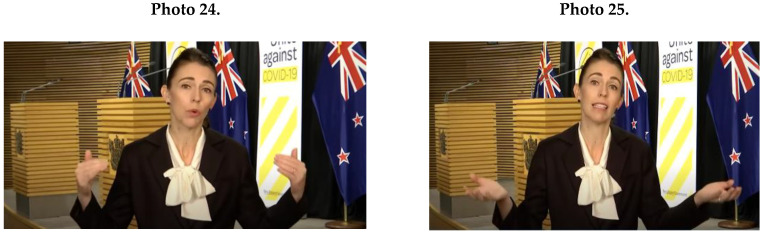


Another masculine pattern used by female leaders (see Figure 1C) was assertive hand movements (*M* = 14.4; *SD* = 5.81), see Photo 26 of Frederiksen.


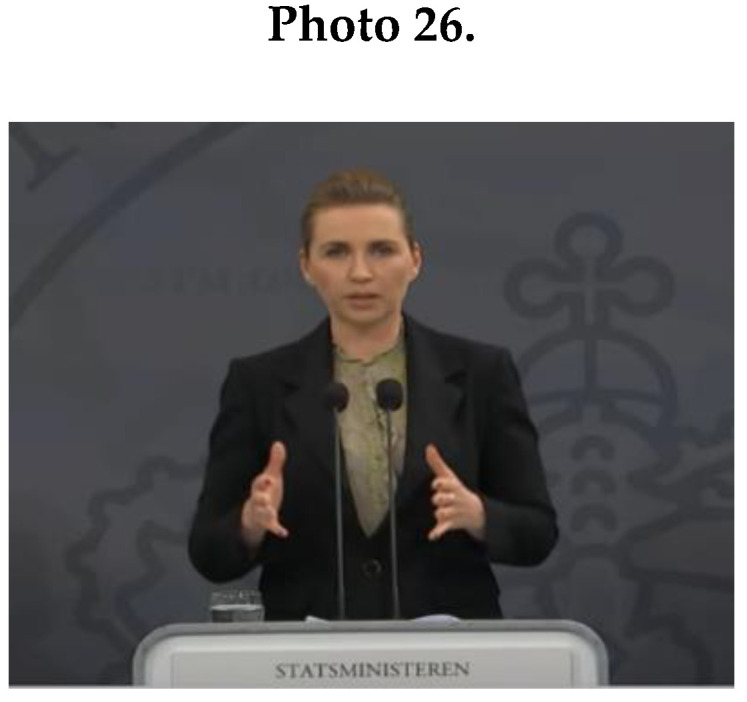


### 3.4. The Interaction Effect of Gender and the Situation of the Pandemic on Political Leaders’ NCS

**Figure 2:**
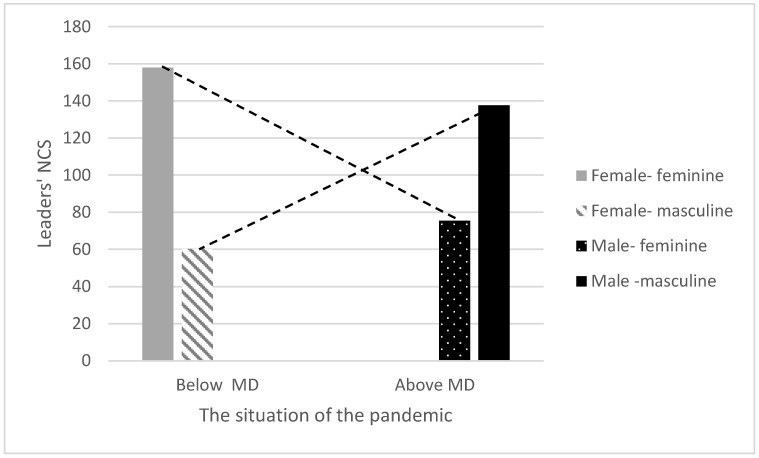
The interaction effect of gender and the situation of the pandemic on leaders’ NCS. (Note. We calculated a median score of the dead/sick ratio to represent the pandemic situation (0.1821) to create a cut-off score, such that below-median scores represent a better pandemic situation, while above-median scores represent a worse pandemic situation.).

Regarding RQ3, an interaction effect was found between gender and the situation of the COVID-19 pandemic in terms of diseased and severe cases on the leaders’ *NCS* (β = 1.12, B = 103.26, t = 3.24, *p* < 0.01). In countries with female leaders, the situation of the pandemic was less severe (that is, few diseased and severe cases), and the leaders’ NCS contained more feminine expressions of certainty, kindness, and optimism during the COVID-19 crisis. However, in countries with male leaders where the situation of the pandemic was more severe (many diseased and severe cases), the leaders’ NCS expressed more masculine manifestations of anger, threatening and warning behaviors, and nonverbal leakage of stress (see Figure 2).

## 4. Discussion

### 4.1. The Effect of Gender on Political Leaders’ NCS

The main conclusions of this study are that gender affects leaders’ NCS. During a crisis, leaders present behavior corresponding to their gendered NCS. These conclusions advance the social construction theory of gender [26] and the role-congruity theory of female leaders [14], which may explain leaders’ gender-corresponding behavior as a means of avoiding the backlash effect that manifests in negative perceptions and evaluations [13]. Previously, a key to political success for female leaders was the performance of masculine NCS [11,21,27]. However, our novel conclusions are that contemporary female leaders do not adopt masculine NCS of leadership; instead, they present a new leadership style based on feminine NCS. Previous research on female leaders focused mainly on periods of political campaigns, while our novel findings of feminine NCS of leadership highlight gender differences in emotions, cognition, and behavior and present an alternative communicative pattern for effective leadership, particularly during a pandemic crisis.

### 4.2. The Effect of Gender on Political Leaders’ NCS of Fear-Arousing

NCS manifestations of fear arousal explain male leaders’ televised appearances during the COVID-19 pandemic. Fear-arousing communication is applied in political communication to change attitudes, perceptions, and behavioral patterns [28]. Persuasion theories have defined the fear factor as the effect of evoking negative emotions and arousing feelings of apprehension or fear, which are considered effective tools in persuasion [29]. However, recent studies of political communication have argued that overly negative and frightening messages can arouse dislike and undermine the credibility of the leader and his/her messages [22]. NCS that arouse fear may provoke antagonism and exacerbate the crisis.

### 4.3. The Effect of Gender on Political Leaders’ Empathic and Optimistic NCS

During the COVID-19 pandemic, emotional NCS explains female leaders’ televised appearances and involves empathetic, optimistic facial expressions, and a compassionate voice. Emotional NCS is attributed to effective political communication and persuasion [7] that has an adaptive meaning to political leaders. Emotional empathic and optimistic NCS conveys messages of security and calmness that puts distance between a challenging, stressful, and frightening situation. During crisis, emotional NCS of empathetic and optimistic expressions mitigates the negative context and helps leaders establish a credible image of self-confidence and control. Viewers prefer leaders who move freely, maintain eye contact, and smile [30], as the persuasive effect emerges from the positive association that enhances positive thinking based on heuristic cues. Emotional communication of empathetic and optimistic NCS is considered an adaptive pattern that characterizes politicians of high standing and winners of political debates [31].

### 4.4. The Effect of Gender on Political Leaders’ Nonverbal Leakage

The explanations for the nonverbal leakage of tension among male political leaders’ NCS in televised appearances during the COVID-19 crisis have emerged from conflictual situations. The situation of a pandemic that involves sickness and death, combined with an economic crisis of unemployment and social and psychological difficulties, creates a conflict between a political leader’s need to present confidence, stability, calmness, and control, and his/her inner state of uncertainty, instability, and helplessness. Also, the goal of preserving the democratic base and human rights conflicts with the goal of presenting declarative statements about fighting the pandemic that restrict freedom of movement and work. Multiple-goal situations of conflictual situations activate assembly difficulties [32]. Feminist theories posit that women are generally better than men at handling conflictual and multifaceted situations and are more willing to embrace complex solutions; this reflects their greater experience with contradictory roles and expectations [20]. Contemporary female politicians manage the challenge of dealing with contradictory expectations from them [33]: they are expected to act as leaders and exhibit masculine NCS, but also to behave like women and display feminine NCS [34].

### 4.5. The Effect of Gender on Political Leaders’ Rational Intentional NCS

Political leaders’ NCS is defined along two axes: rational versus emotional, and intentional versus unintentional. During the COVID-19 crisis, male political leaders mainly expressed rational and intentional NCS that represent propositional content of symbolic patterns [35], which helps clarify messages and improves understanding. Leaders’ rational, intentional, illustrative gestures enhance the perceptions of the messages and increase the viewers’ attention, promote memory, and enhance recalling processes. Persuasion based on reasoning is the central route, which encourages a systematic process of thinking through an issue [36]. Male leaders expressed masculine, rational, intentional NCS centered on reasoning, facts, and logical attitudes during the complex crisis.

### 4.6. The Effect of Gender and the Situation of the Pandemic on Political Leaders’ Maculine/Feminine NCS

This study has presented a theoretical and analytical framework of political leaders’ gendered NCS during crisis, and established major conclusions regarding the novel profiles for charismatic leaders and effective political communication. The COVID-19 pandemic has generated novel feminine NCS to establish public cooperation, trust, confidence, and engagement. The COVID-19 crisis that the world is currently facing has moved the male-dominated, masculine NCS—which mainly expresses competition, threatening behavior, extroverting gestures of broad proxemics, tension leakage, anger, and fear—to one side, and presents an alternative feminine NCS for leadership.

The new feminine NCS of female political leaders expresses cooperation and coordination, emotional communication, extensive facial expressions, an expressive voice, flexible expressions, optimistic, and gentle, calm NCS. Interestingly, the effect of gender on leaders’ NCS also had an interaction effect with the situation of the pandemic. Thus, in countries with female leaders, the situation of the pandemic was minor (relatively few diseased and severe cases), and the leaders’ NCS displayed nonverbal feminine expressions of certainty, kindness, and optimism.

### 4.7. Summary and Additional Avenues for Future Research

This study delineates the gendered NCS of political leaders’ televised appearances during the COVID-19 crisis and develops theoretical and analytical frameworks of the unique gendered NCS of male versus female leaders. The contemporary crisis presents feminine NCS for leadership that provides an alternative to the masculine-dominated political NCS. The novel feminine NCS expresses emotional communication, flexible expressions, calmness, cooperation, and optimism, in contrast to the masculine NCS of competition, threatening behavior, tension leakage, and fear. The novel feminine NCS replaces the common perception that a woman who wishes to reach top leadership positions must adjust to masculine norms and behavioral codes.

The presented theoretical and analytical framework may offer additional avenues for future research into gender and political NCS during a crisis. Future research could adopt our framework to examine political leaders’ NCS from other countries, particularly in non-democratic countries, and to analyze NCS of health/economic experts.

## 5. Conclusions

Finally, the conclusions from this study could have meaningful practical implications for male and female political leaders. Political leaders may adopt the proposed theoretical and analytical framework to develop and improve their communication skills, persuasion effects, social influence, public support, and cooperation into political success.

## Figures and Tables

**Table 1:** Research questions.

No.	Research Questions
**RQ1.**	What has been the gender effect of females vs. males on leaders’ NCS during the COVID-19 crisis?
**RQ2.**	Have female vs. male leaders expressed feminine or masculine NCS during the COVID-19 crisis?
**RQ3.**	Has the effect of gender on leaders’ NCS had an interaction effect with the situation of the pandemic (in terms of diseased and severe cases)?

**Table 2:** Male/female political leaders’ televised appearances and the situation of the pandemic.

Country	Population	Leader’s Name	Date of Televised Appearances	Confirmed Cases	Deaths
		**Male political leaders**			
United States	328 million	Donald Trump	31 March 2020	17,987	2398
			10 April 2020	30,869	14,665
United Kingdom	66 million	Boris Johnson	16 March 2020	251	43
			22 March 2020	1035	250
Israel	9 million	Benjamin Netanyahu	14 March 2020	52	0
			1 April 2020	298	21
Italy	60 million	Giuseppe Conte	22 March 2020	6557	4827
			9 April 2020	3636	17,669
Canada	37 million	Justin Trudeau	17 March 2020	120	1
			11 April 2020	1467	531
		**Female political leaders**			
Belgium	11 million	Sophie Wilmès	12 March 2020	174	5
			3 April 2020		
Finland	5.5 million	Sanna Marin	25 March 2020	92	1
			8 April 2020		
Denmark	5.7 million	Mette Frederiksen	23 March 2020	69	13
			6 April 2020	292	179
New Zealand	4.9 million	Jacinda Ardern	22 March 2020	13	0
			29 March 2020	60	1
Germany	83 million	Angela Merkel	19 March 2020	2801	20
			9 April 2020	4974	2107

Note. Population data according to the World Bank; confirmed cases and death data according to the World Health Organization (WHO).

**Table 3:** Gendered Nonverbal Communicative Structure (NCS) and Cohen’s Kappa Reliabilities.

Masculine Nonverbal Communication		Feminine Nonverbal Communication	
Communicative Patterns	Cohen’s Kappa	Communicative Patterns	Cohen’s Kappa
Angry facial expressions	0.93	Expressive facial expression of Helplessness	0.90
Stable and monotonous speech	0.90	Small movements	0.91
Ascending posture	0.91	Hesitation/fluency problems	0.92
Clenched fists	0.92	Enclosing posture	0.92
Deep voice	0.87	Round movements	0.90
Loud voice/shouting	0.91	Diverse intonation	0.89
Tension leakage of side by side movements/licking of lips	0.92	Rapid voice	0.92
Illustrative gestures	0.89	Expressive voice	0.89
Broad hand movements/broad proxemics	0.90	Smiling	0.91
Assertive hand movements	0.94	Descending posture	0.91
Sharp movements/displaying anger	0.93	Making eye contact	0.90
Gestures of warning and threat	0.89	Surprised facial expression	0.90

## Appendix A

Attached is the coding procedure of gendered NCS pattern descriptions and exemplifications. The coding procedure is a multi-tiered system for observed nonverbal communication classification, which includes gestures, postures, facial expressions, vocalic characteristics, and performance. This multi-tiered system contains NCS patterns that are cumulative categories and are not exclusive, so each nonverbal communication structure includes varies gendered patterns.

**Table A1:** Gendered NCS Patterns: Descriptions and Exemplifications.

Gendered NCS Pattern	Description and Exemplifications
	**Masculine Nonverbal Communication**
Angry facial expressions	Anger is shown in the face when the eyebrows are pulled down, upper eyelids pulled up, lower eyelids pulled up, margins of lips rolled in, lips may be tightened.
Stable and monotonous speech	Continuous speech, uniform in pitch or inflection. An example is an unvarying tone, marked by a sameness of intonation and intensity.
Ascending posture	A straightforward position of the body, when the upper body is held up straight.
Clenched fists	A hand gesture in which the fingers are clenched in the palm.
Deep voice	A voice that is low in pitch.
Loud voice/shouting	Speech of relatively loud volume.
Tension leakage	A physical movement that indicates stress; for example, side by side movements or licking of lips.
Illustrative gestures	A gesture that provides a visual image of what is being said verbally; for example, saying “up” and raising one’s hand up.
Broad hand movements/broad proxemics	A use of large physical space; for example, a large hand gesture that spreads over a large space.
Assertive hand movements	A hand movement that is held still under control at a steady rate; for example, a finger-wagging gesture, the movement of the index finger from left to right to left, as if to say no or reject something.
Sharp movements/displaying anger	A beating gesture or a hand gesture displaying anger; for example, up-and-down or back-and-forth hand movements that coincide with spoken clauses, breaks, or sentence ends.
Gestures of warning and threat	For example, the movement of the index finger up and down and up, such as to threaten or reprimand someone.
	**Feminine Nonverbal Communication**
Expressive facial expression of helplessness	A face expression that expresses that there is nothing that anyone can do to improve a bad situation, and that control over the situation or its outcomes is impossible.
Small movements	A small movement, such as a minor hand gesture, which does not take over a large space.
Hesitation/fluency problems	Problems with the flow, rhythm, and speed, meaning that speech may sound interrupted or blocked. For example, repeating part or all of a word; dragging out syllables; talking breathlessly, merging words together or cutting off parts of them; saying “um” or “uh” often when talking.
Enclosing posture	A closed body position, such as crossed arms or hands held together.
Round movements	A circular motion, a movement of rotation; for example, a metaphoric hand gesture, movements of the hands that represent or indicate the source domain of a metaphor.
Diverse intonation	A variation in pitch of speech, or rise and fall in pitch of the voice in speech. A variation in tone of speech.
Rapid voice	Fast talking and a high rate of speech. Pressured speech that contained no pauses or stops.
Expressive voice	An expressive voice pauses and quickens; changes pace, lowers and raises both volume and pitch. It carries emotion expression by varying the elements of sound: volume, pitch, rhythm, and timbre.
Smiling	Facial expression of happiness, when the muscles around the eyes tighten, “crow’s feet” wrinkles around the eyes, cheeks raised, lip corners raised diagonally.
Descending posture	A curved position of the body, when the upper body is bent, joined by the upper cervical spine. Ipsilateral lower neck, shoulder and contralateral upper back and ipsilateral lower back muscles.
Making eye contact	Visual contact in which the speaker looks directly into the other’s eyes.
Surprised facial expression	Facial expression of surprise, when the entire eyebrow is pulled up, eyelids pulled up, mouth hangs open, pupils dilated.
